# Supplementary material for: Advancing the representation of reservoir hydropower in energy systems modelling: The case of Zambesi River Basin
Source: PLoS One. 2021 Dec 2;16(12):e0259876. doi: 10.1371/journal.pone.0259876 (PMC8638992; doi:10.1371/journal.pone.0259876)
Supplement: S1 File — (DOCX) [file pone.0259876.s001.docx]

Advancing the representation of Reservoir Hydropower in Energy Systems Modelling: the case of Zambesi River Basin

*Nicolò Stevanato ^a,b,*^, Matteo V. Rocco ^a^, Matteo Giuliani ^c^, Andrea Castelletti ^c^, Emanuela Colombo ^a^*

(a) Department of Energy, Politecnico di Milano, Milan, Italy

(b) Fondazione Eni Enrico Mattei, Milan, Italy

(c) Department of Electronics, Information, and Bioengineering, Politecnico di Milano, Milan, Italy

* Corresponding author: N. Stevanato, Tel.: +39-02-2399-3866; address: Via Lambruschini 4, 20156 Milan, Italy. E-mail: [nicolo.stevanato@polimi.it](mailto:matteovincenzo.rocco@polimi.it)

# Technoeconomic Parameters

Table SI 1. Technoeconomic parameters of the modelled technologies

| Power Plant Category | Lifetime [years] | Conversion Efficiency [-] | Investment Cost [USD/kW] | O&M Fixed [USD/kW-year] | O&M Variables [USD/kWh_el] | Fuel Cost [USD/kWh_th] |
| --- | --- | --- | --- | --- | --- | --- |
| Diesel Engine | 30 | 0.35 | 708 | 24 | 0.0030 | 0.049 |
| Gas Engine | 30 | 0.40 | 708 | 24 | 0.0024 | 0.011 |
| OCGT | 30 | 0.30 | 708 | 24 | 0.0024 | 0.049 |
| Coal | 50 | 0.35 | 1800 | 32 | 0.0024 | 0.007 |
| Coal Old | 50 | 0.20 | 1800 | 32 | 0.0024 | 0.007 |
| HFO | 50 | 0.35 | 1350 | - | 0.015 | 0.014 |
| Nuclear | 60 | 0.33 | 4480 | 112 | - | 0.004 |
| Biomass | 30 | 0.40 | 4325 | 54 | 0.003 | - |
| PV | 25 | - | 1158 | 12 | - | - |
| Wind | 25 | - | 1940 | 37 | - | - |
| Hydro | 30 | - | 1815 | 48 | - | - |

# Country Modelled Plants

Table SI 2. Technologies modelled in each country

| Country | Technology | Capacity [MW] |
| --- | --- | --- |
| Botswana | Coal | 732 |
|  | OCGT | 90 |
|  | Diesel Engine | 70 |
| Mozambique (N-C) | Cahora Bassa | 2’075 |
|  | Diesel Engine | 0.12 |
|  | OCGT | 14 |
| Mozambique (S) | Hydro | 16.6 |
|  | Gas Engine | 499.6 |
|  | OCGT | 24 |
| Namibia | Coal | 120 |
|  | Diesel Engine | 38.5 |
|  | Hydro | 300 |
| South Africa | Coal | 37’868 |
|  | Nuclear | 1’860 |
|  | OCGT | 3’409 |
|  | PV | 1’474 |
|  | Wind | 2’078 |
| Zambia | Ithezi Thezi | 120 |
|  | Kafue Gorge | 990 |
|  | Kariba (N) | 1’080 |
|  | Hydro | 207.5 |
|  | Diesel Engine | 88.88 |
|  | HFO | 110 |
|  | Coal | 300 |
|  | PV | 1 |
| Zimbabwe | Kariba (S) | 750 |
|  | Coal | 920 |
|  | Coal Old | 270 |
|  | Biomass | 96.5 |

# Dispatch Strategy Results

- 1. Calliope_Base


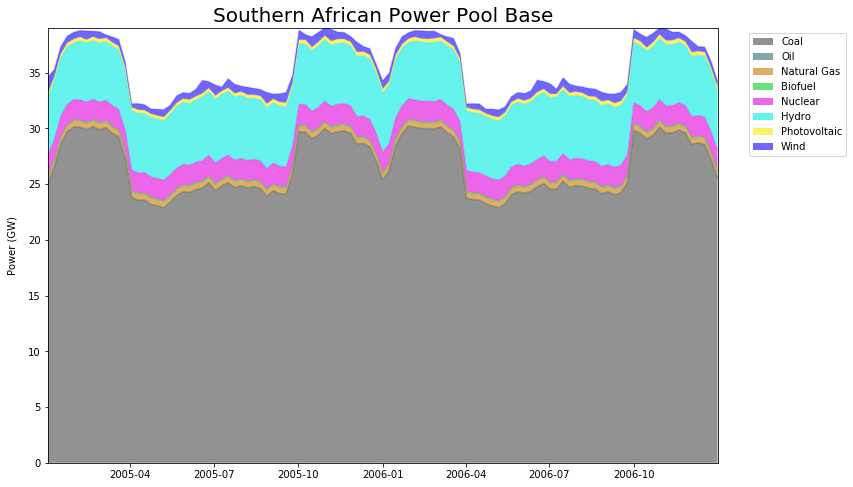


Figure SI 1. SAPP Dispatch strategy as result of Calliope Base


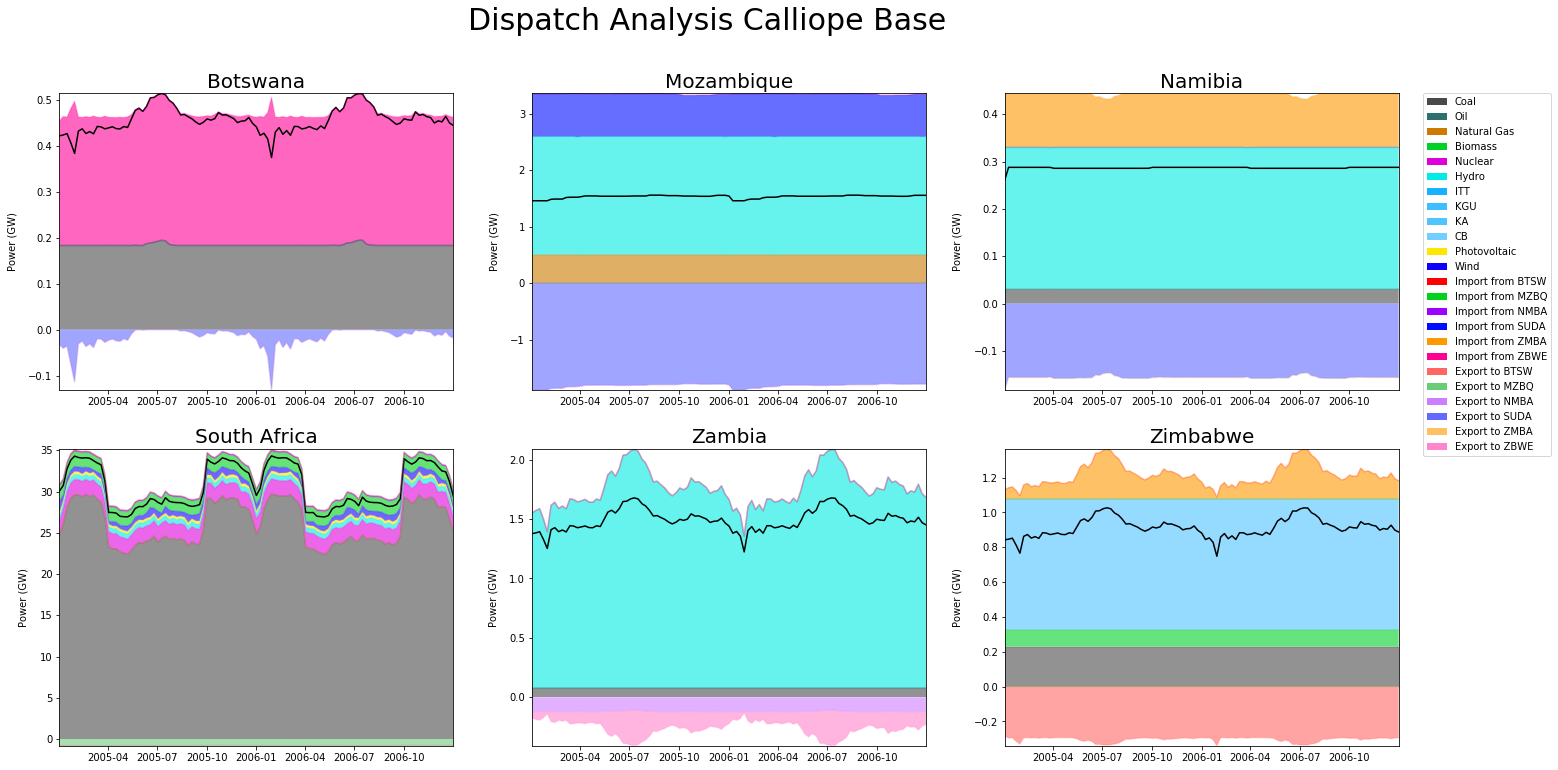


Figure SI 2. Country by Country SAPP Dispatch strategy as result of Calliope Base

- 1. Calliope_Hydro


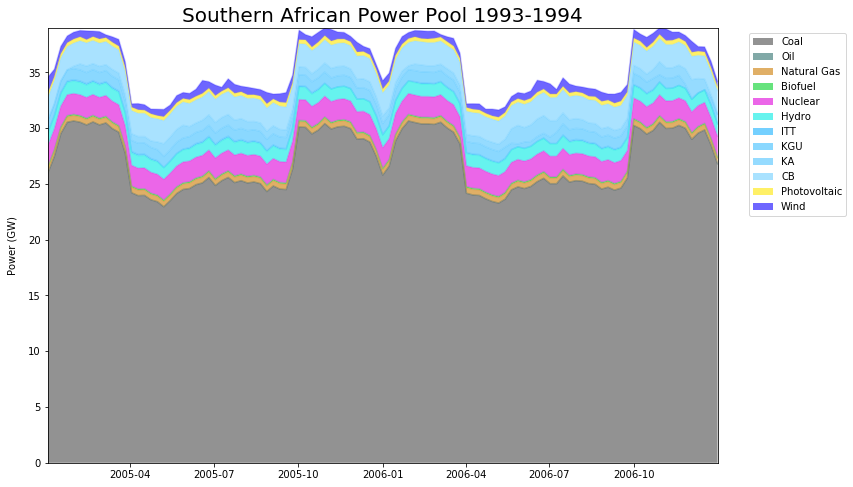


Figure SI 3. SAPP Dispatch strategy as result of Calliope Hydro Scenario 1993-1994


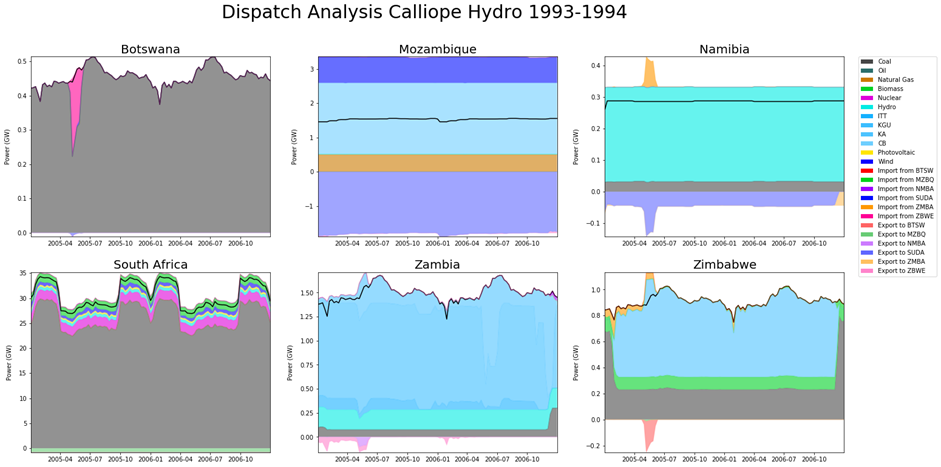


Figure SI 4. Country by Country SAPP Dispatch strategy as result of Calliope Hydro Scenario 1993-1994


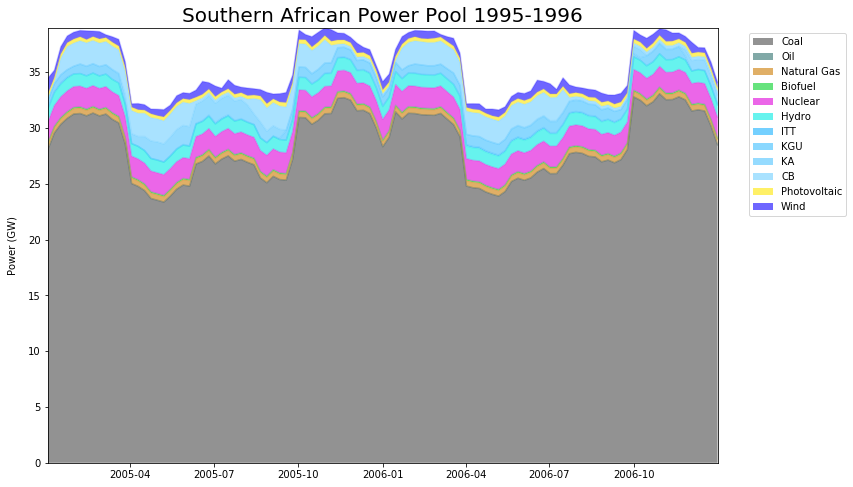


Figure SI 5. SAPP Dispatch strategy as result of Calliope Hydro Scenario 1995-1996


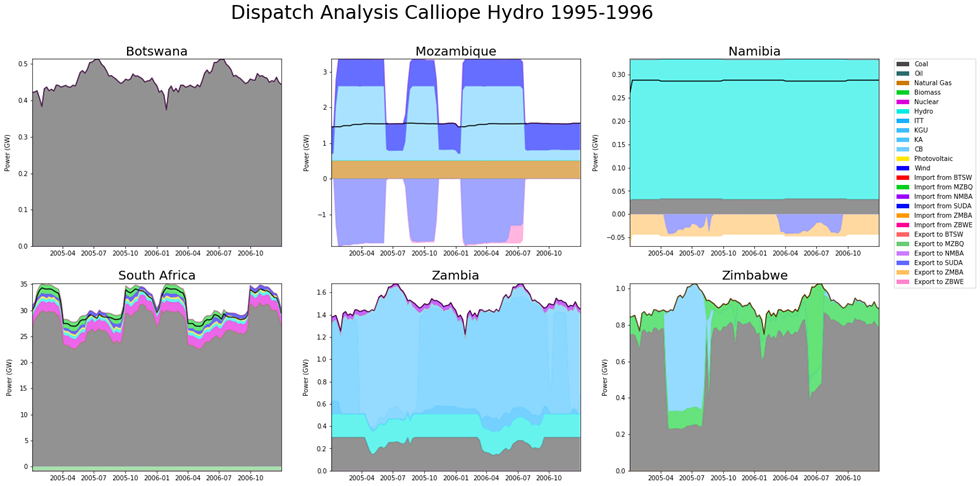


Figure SI 6. Country by Country SAPP Dispatch strategy as result of Calliope Hydro Scenario 1995-1996


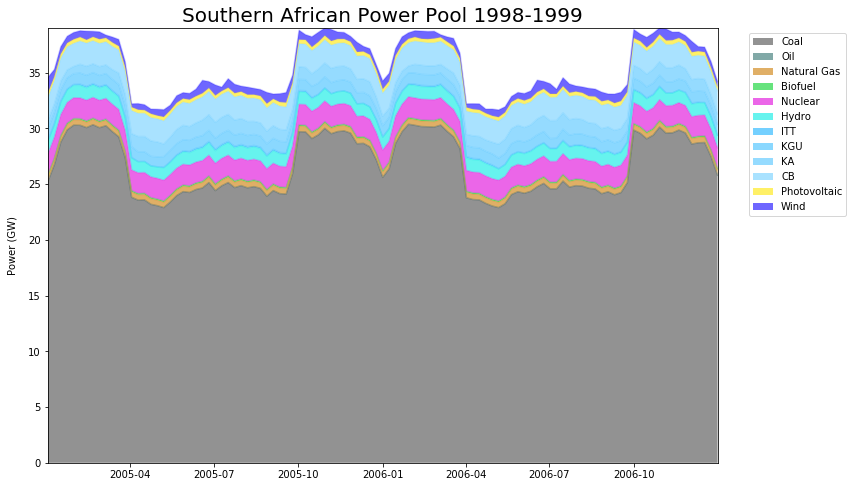


Figure SI 7. SAPP Dispatch strategy as result of Calliope Hydro Scenario 1998-1999


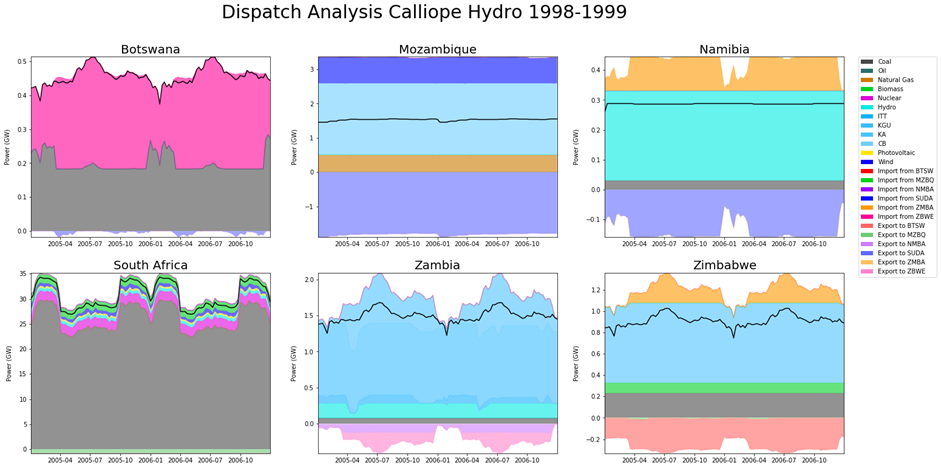


Figure SI 8. Country by Country SAPP Dispatch strategy as result of Calliope Hydro Scenario 1998-1999


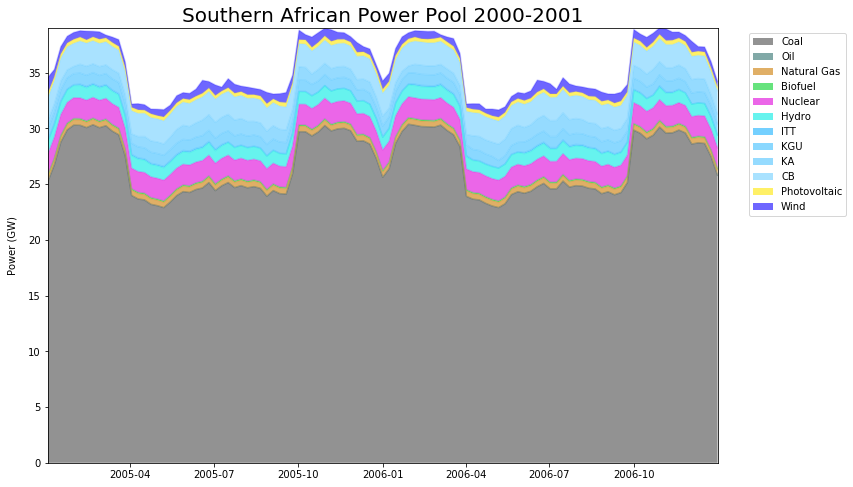


Figure SI 9. SAPP Dispatch strategy as result of Calliope Hydro Scenario 2000-2001


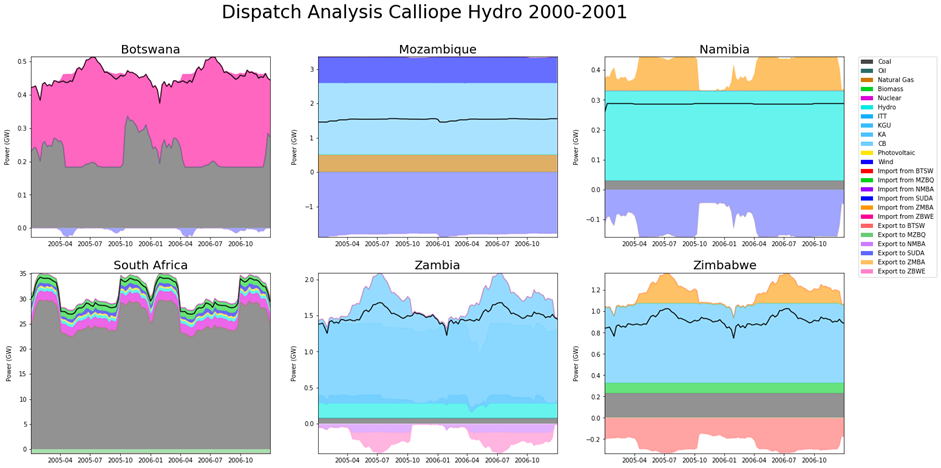


Figure SI 10. Country by Country SAPP Dispatch strategy as result of Calliope Hydro Scenario 2000-2001

- 1. Differential Dispatches


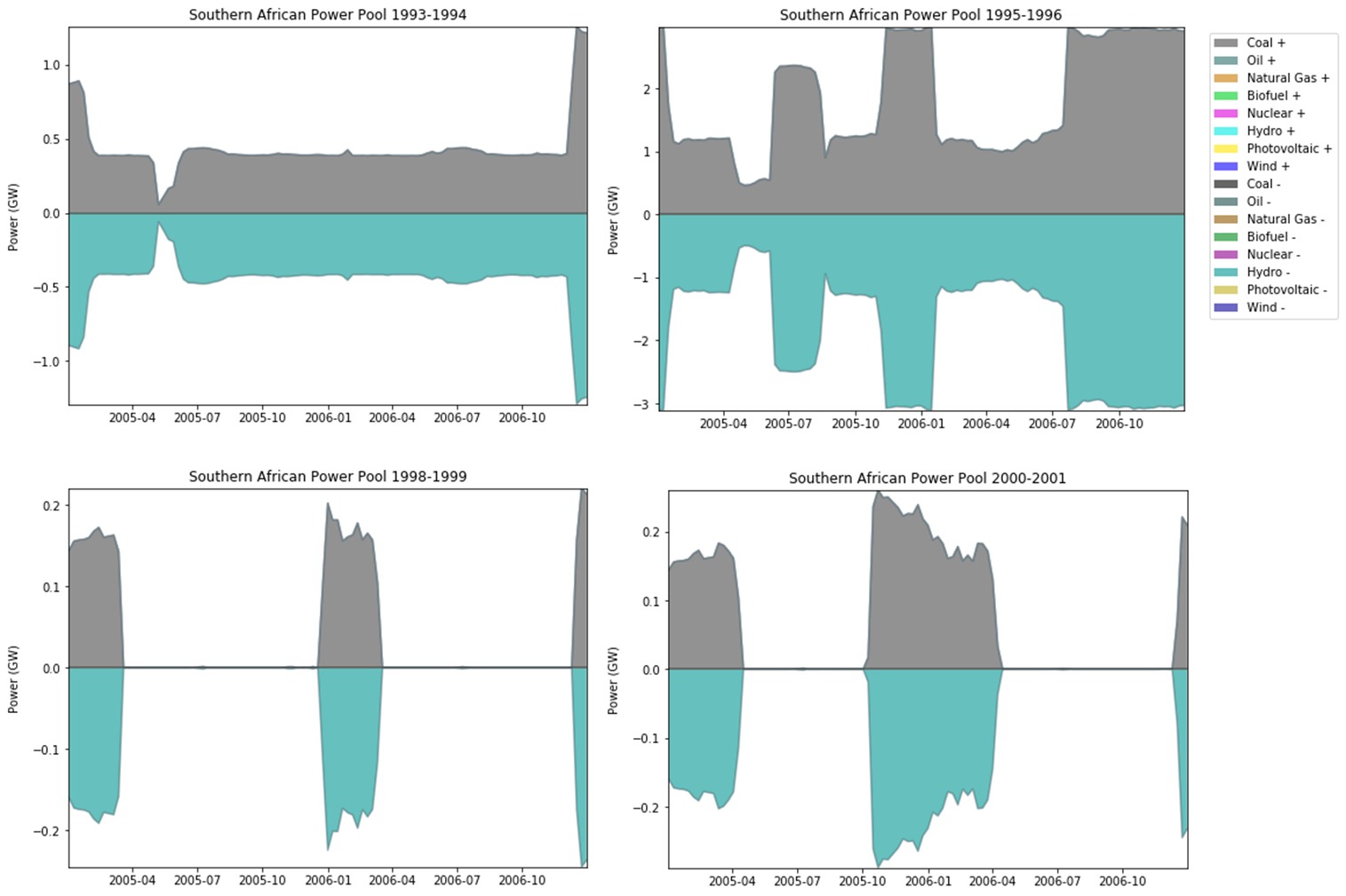


Figure SI 11. SAPP Differential Dispatch, Hydro vs Base in the four scenarios


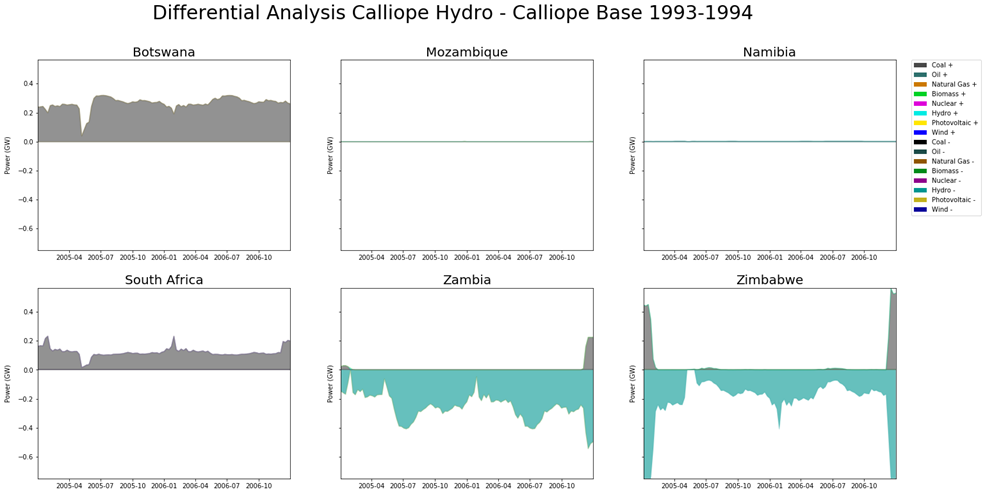


Figure SI 12. Country by Country SAPP Differential Dispatch, Hydro 1993-1994 vs Base


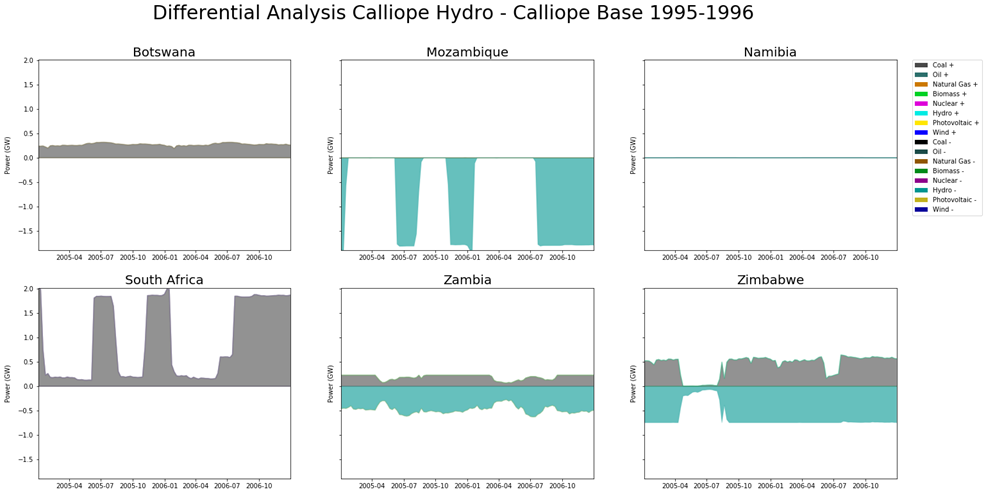


Figure SI 13. Country by Country SAPP Differential Dispatch, Hydro 1995-1996 vs Base


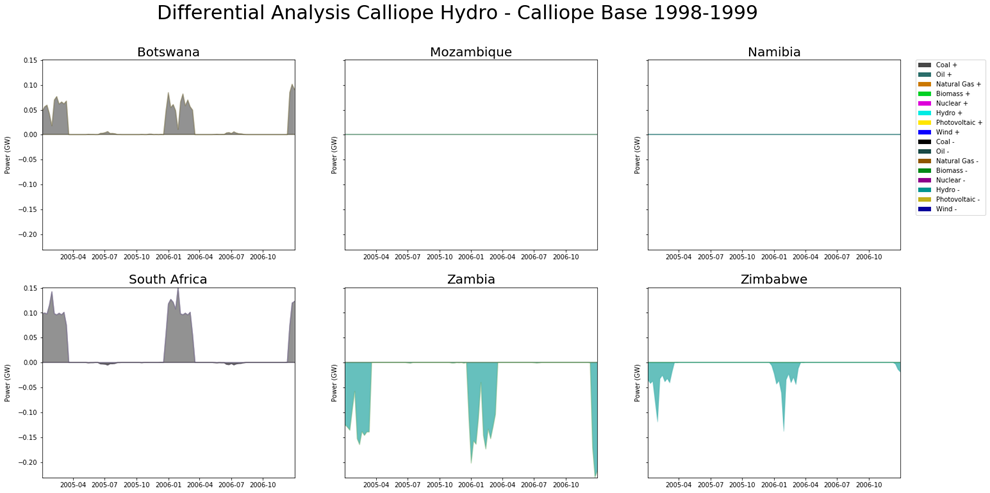


Figure SI 14. Country by Country SAPP Differential Dispatch, Hydro 1998-1999 vs Base


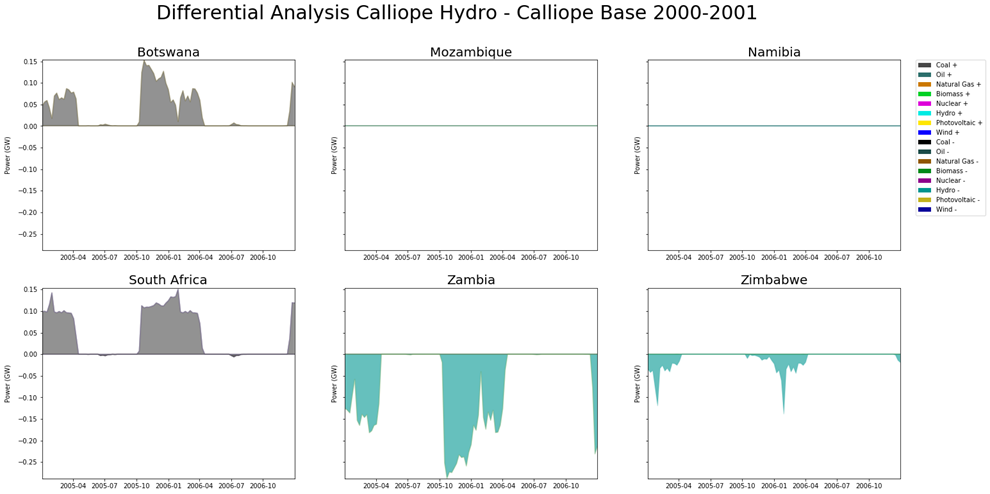


Figure SI 15. Country by Country SAPP Differential Dispatch, Hydro 2000-2001 vs Base
