# Supplementary material for: Advancing the representation of reservoir hydropower in energy systems modelling: The case of Zambesi River Basin
Source: PLoS One. 2021 Dec 2;16(12):e0259876. doi: 10.1371/journal.pone.0259876 (PMC8638992; doi:10.1371/journal.pone.0259876)
Supplement: S1 Appendix — (DOCX) [file pone.0259876.s002.docx]

# Acronyms

OSeMOSYS Open Source energy MOdelling SYStem

SAPP Southern African Power Pool

WAPP West African Power Pool

ZRB Zambezi River Basin

# Symbols

$\dot{E}_{e}$ electrical power

$\dot{m}_{w}$ water flow rate

$g$ gravity acceleration

$H$ hydraulic head

$\eta_{e}$ efficiency of the turbine

$ev_{loss}$ evaporation losses

$ev_{coeff}$ evaporation coefficient

$S_{t}$ reservoir surface

$S_{loss}$ storage losses

$S_{cap}$ storage capacity
