# Supplementary material for: Advancing the representation of reservoir hydropower in energy systems modelling: The case of Zambesi River Basin
Source: PLoS One. 2021 Dec 2;16(12):e0259876. doi: 10.1371/journal.pone.0259876 (PMC8638992; doi:10.1371/journal.pone.0259876)
Supplement: Attachment — Submitted filename: Response to Reviewers_2ndRev.docx [file pone.0259876.s004.docx]

**Ref: PONE-D-20-40043**

**Title: Advancing the representation of reservoir hydropower in energy systems modelling: the case of Zambesi River Basin**

**Journal: PLoS One**

**Response to Reviewers:**

**EDITOR COMMENTS:**

***1.*** *As both reviewers stress, the literature review is still not well written. It requires a major rewrite.*

The literature review has been substantially restructured and re-written. We hope it now meets the quality standards required.

***2****. Likewise, the contribution of your study remains unclear. It even seems more unclear than before, as the scope of the study was clarified to be much more limited than it seemed to be.*

We hope that by improving the literature review and changing the wording of the “aim of the work” section, this is now clearer.

***3.*** Make assumptions and what is out of scope transparent and critically discuss the limitations of your study.

*Following Reviewers’ comments we stated every assumption and limitation of the work.*

***4.*** *As pointed out earlier, let a native speaker carefully proofread the manuscript.*

We have increased our efforts to improve the English throughout the manuscript. But the Editor still feels that we should pursue professional proofreading we can do so.

**REVIEWER #1 COMMENTS:**

***1.*** *Title: Please use small letters with "reservoir," "hydropower," "energy," "systems," and "modelling."*

It has been corrected.

***2.*** *Line 21: since you modified the scope of the paper contribution, please add the word "energy" before "modelling framework".*

Thanks for pointing out. It has been inserted.

***3.*** *Why is the literature review section number 1.1 and not 2, while the introduction is 1. This is not clear.*

Please consider that the aim of the work is not the literature review, for this reason the review of previous works on the matter was considered to be part of the introduction, as a way to frame the intervention in the broader picture. For this reason, the literature review was not dedicated its own chapter. For the same reason, and according to the following comments and comments form Reviewer #2, we have changed name and scope of the subchapters, without conducting a full-fledged literature review. We restructured chapter 1 as follows:

1.1 Energy and Water
1.2 Modelling hydrological and energy systems
1.3 Representation of hydropower in energy modelling
1.4 Aim of the work

We believe this way the scope of the work is better framed and justified.

***4.*** *The literature review section is still very confusing. The authors attempted to address my previous comment by adding a paragraph to the beginning of the section. This is good, but not enough. The section needs a full rewrite. Breaking down the section into sub-sections might help remove some of the confusion.*

This comment helped in reshaping Chapter 1, see answer to previous comment for details.

***5.*** *Table 1: what is a "New model" integration?*

The term “New Model” was meant to identify an alternative to the integration of two models, being in fact the creation of a new model from scratch. We decided to remove Table 1 from the manuscript as it was drawing too much attention on the previous modelling efforts, while the aim of the work is not to conduct a literature review. The identification of previous efforts is only instrumental to provide the background knowledge to the reader to fully capture the value of our proposed approach.

***6.*** *Aim of the work: the contribution is still ambiguous. Why don't the authors explicitly state which open-source model they are trying to improve instead of saying, "improving the representation of reservoir hydropower within open-source energy systems modelling" and "improving the way reservoir hydropower is represented in existing energy system models." You need to be clear here that you are improving a certain open-source modelling tool.*

In line 217 we state that the model we are using as proof of concept is Calliope, but the validity of the approach is wider that the single case of Calliope. Indeed, every energy modelling framework defined based on power nodes (1) could potentially benefit from the proposed approach.

(1) (Heussen, K. et al., 2010. Energy storage in power system operation: The power nodes modelling framework. In Innovative Smart Grid Technologies Conference Europe (ISGT Europe), 2010 IEEE PES. pp. 1–8. DOI: 10.1109/ISGTEUROPE.2010.5638865).

***7.*** *Aim of the work: the scope of the work is to improve the representation of hydropower in an open-source energy modelling tool. That is fine, but how does this compare to integrated water-energy modeling approaches? Should the science and practice drop the idea of integrated water-energy modeling and try to improve hydropower representation in energy models? What I am missing here is a bridge between the literature review and the study contribution. It would be best to highlight how your work provides added value compared to previous approaches, including integrated water-energy modeling. This is a major issue that needs to be addressed. If the authors cannot find an added value of their proposed approach compared to comprehensive water-energy modeling, they should focus the paper around the case study rather than the modeling approach.*

Thanks for the comment, it allowed us to better frame our contribution:

Lines 138-150: *“The issue with the presented cases is the lack of replicability of those integrated energy-water frameworks. The mentioned approaches are indeed rarely replicated to other geographical areas, given the complex structure of the integrated models, which are sometimes ad-hoc developed around a specific case study. The scientific value of such integrated models is indeed relevant and not questioned in this work, which is instead focused on model replicability in broader engineering applications.*

*On the other hand, most energy system models (both proprietary and open-source) are adopted as a standard by the international community [35] and used in a wide range of applications, as discussed later in section ‘Aim of the work’. However, their representation of a variety of engineering phenomena, including hydropower, leave space for improvements. Therefore, improving an already existing energy modelling framework may have a broader impact on the international community in terms of model accessibility by users and replicability of the analysis in other contexts.*

*Given these considerations, this work proposes a methodology for improving the reservoir hydropower representation in open-source energy system models.”*

***8.*** *The issue of aggregated country-level energy demand nodes needs to be highlighted as a limitation, and its implications for the results need to be discussed.*

In modelling energy systems, the level of aggregation of country energy demand depends by the aim and scope of the analysis and, secondly, the availability of detailed energy demand data.

For example, if the analysis aims to investigate the reliability of transmission and distribution network within a country, then a single-node model would be not appropriate. The focus of the proposed application is instead in quantifying the effects of a better characterization of the hydropower system in a multi-regional context, assessing the changes in national supply technology mixes.

Given the complexity of national energy systems and the non-homogenous distribution of natural resources and final energy demand, relying on multi-nodal representation of each country may provide more detailed and accurate results. However, the paucity and high uncertainty of raw energy data in the sub-Saharan region must be properly considered in order to avoid that on overly detailed model relying low quality data results in an uncontrolled error propagation.

Finally, please consider the following relevant and recent literature references, adopting single-region energy models applied in the African context.

Pavičević, De Felice, Busch, Hidalgo González, Quoilin, *Water-energy nexus in African power pools - The Dispa-SET Africa model*, Energy, Volume 228, 2021, 120623, <https://doi.org/10.1016/j.energy.2021.120623>

Taliotis, Shivakumar, Ramos, Howells, Mentis, Sridharan, Broad, Mofor*, An indicative analysis of investment opportunities in the African electricity supply sector — Using TEMBA (The Electricity Model Base for Africa)*, Energy for Sustainable Development, Volume 31, 2016, Pages 50-66, <https://doi.org/10.1016/j.esd.2015.12.001>

In light of this, Lines 465-472 were added: “A further limitation of the modelling approach is the single node representation of the energy demand of each country, exception made for Mozambique, characterized by two demand nodes. In energy system modelling, the level of aggregation of country energy demand depends by the aim and scope of the analysis and, secondly, the availability of detailed energy demand data. Increasing the number of demand nodes characterization may have resulted in more detailed and accurate results. For the context of analysis, one demand node per country was considered a fair trade-off between the data paucity that characterizes the Sub-Saharan context and results accuracy. This choice is anyway in line with the most recent literature on the subject [72,73].”

***9.*** *Water use for purposes other than hydropower is not included in the case study analysis. This is a major issue. It would be helpful if the authors could attempt to add these other uses. Or at least discuss this limitation in more detail, including its implications for the presented results.*

We agree that the use of water for other purposes is an important aspect, and it was in fact included in the “Current Limitations” section. We discussed the implications of this limitation further in the manuscript.

Line 369-375: *“Not considering the non-hydropower uses of water implies that the amount of water that appears to be available for the turbines according to the model is actually more than the amount available in real life. The main other use of water in the area is water for agriculture, that is extracted directly from the river basin and from the dams, the water used for agriculture should hence be subtracted from the amount of water flowing in the system. This could be modelled in the framework by inserting, as stated above, a demand for non-hydropower water, that has to be satisfied by the system; that water would then be removed from the system.”*

Line 614-616: *“It is worth noting that including in the analysis also non-hydropower uses of water, would shift even more in this results towards a lower availability of hydropower in the system, given the fact that considering it would decrease the amount of water available in the system for power production purposes.”*

***10.*** *Previous comment number 34: the response does not make sense. The authors use historical data to validate the results of their approach. However, their SAPP model assumes a single market that did not exist in that historical period. I am failing to understand the logic here, but maybe I am missing something.*

To test the effects that different water inflow scenarios have on energy supply dispatch, we selected real water inflow scenarios, ranging from wetter to dryer, based on empirically observed data in different past years (some of which are 20 years old). We selected those data with the sole purpose of relying on realistic inflow patterns.

As specified in the reply to your past comment #34, the aim of this case study is to test the change in behaviour of the model according to an improved modelling approach for the hydropower system, relying on different hydrological inflow profiles. The nature of the power system, either single or open energy market is not relevant for the current application, nor hindering the validity of the obtained results. This is in accordance with the common practice in energy system modelling for multi-regional cases, where the model works based on a single, perfectly informed energy regulatory authority, which minimizes the overall operational costs of energy generation considering the entire area as a single market.

**REVIEWER #2 COMMENTS:**

***General:*** *As it seems that data is available for Africa, I wounder why the manuscript is limited to such a small case-study.*

Because it was interesting to focus on the inter-national dynamics of electricity exchange in a basin of sub-continental relevance, enlarging the analysis to other basins would not add anything to the methodology, the main contribution of the work; the case study is only meant to show an application. In addition, Africa is not all interconnected, the analysis of the entire continent would just result in a series of smaller isolated sub-continental power pools, like the SAPP analysed in this case.

***10.*** *Author comment :We changed the sentence as follows: “climate variability (i.e. more frequent and intense droughts and floods) and allocations to other uses (e.g. irrigation, domestic and industrial water supply, ecosystem preservation)” Lines 52-54*

*Reviewer reply (R) : so in the end climate variability means precipitation? Does an increase in precipitation always directly lead to more floods?*

The sentence has been expanded into:

*“Hydropower generation is largely dependent upon climate variability, which may either curtail production during intense drought events that reduce water availability [14] or induce large water losses as a consequence of spilling during flood events [15]. In addition, hydropower production is also influenced by other competing water users such as irrigation, domestic and industrial water supply, and ecosystem preservation (e.g., 40% of existing hydropower dams serve multiple demands [16])”* Lines: 53-57

***12.*** *Author comment : Line 59: Structure of the introduction: So far you have mentioned the research gap but not what the aim of the study is, but now I am presented with a chapter “Literature review”. Why has this been done, why is it important? Thanks for the comment. The relevance of the literature review has been made explicit. Lines 65-66: “An extensive review of literature concerning modelling energy and hydrological interdependencies related to hydropower is carried out in order to assess how past works delt with the issue.”*

*R: I feel sorry to say that, but for me personally the introduction section structure could still be improved further.*

*In section 1 you end with what current models do not account for.*

*and in section 1.1 you start with “delt with the issue”*

*But what I think is mentioned is: what is the consequence or issue of the research gap presented in section 1?*

The entire Chapter 1 has been restructured, expanded and overall improved to better frame our intervention in the background identified, according to this comment, following comments on literature review and Reviewer #1 comments on the subject.

***17.*** *Author comment : Line 169: Based on what criteria have you selected/ reviewed the literature? Is this overview meant to be comprehensive? More information is need Thanks for highlighting this shortcoming, the required information have been added. Lines 69-71: “The review of literature is conducted by selecting the most relevant works among the results of a query in Google Scholar under the keywords “Energy Modelling”, “Hydro Modelling”, “Reservoir Hydropower”, selecting works representing the six categories listed above*

*R: So you used “” which, retrieves an exact match of phrase, without any wildcard. So what about : Modelling (UK spelling) vs modeling (Us spelling). So you only searched for for papers with UK spelling? With “reservoir hydropower” you will not find “model for hydropower reservoirs”*

*How many papers have you assessed to get Table 1?*

*You now just simply added the 2 publications mentioned by reviewer 1 in a new category. That contradicts your methods of the literature review. Would you find them with the used key-words? Or would that require a change of key-words?*

*I would like to refer to Reviewer 1, Comment 1, and also ask Reviewer 1 once more to check for comprehensives of the Literature review, considering the search key-words.*

*In lines 65-99 you introduce 6 model categories. Wouldn’t it be good to refer to the same categories in Table 1?*

The entire structure of chapter 1 has been changed, and the reviewed publications do not constitute a “Literature Review” anymore, but are just used to frame our intervention in a background that justifies the relevance of the approach. Please consider that a literature review is not the goal of the paper, and the attention of the reader should be focused on the methodology.

Table 1 does has been removed as it was not relevant to introduce the aim of the work.

***21a.*** *Author comment: Section 2.1 defines the methodological strategy that transforms Calliope_Base into Calliope_Hydro, while section 3.1 describes the scope and assumptions of the Zambezi River Basin and SAPP case study implemented in the new Calliope_Hydro. Both sections were partially restructured and re-written to better highlight this.*

*R: According to the track changes you added 1 sentence in section 2.1 and 2 in section 3.1. Can we consider this as restructured and re-written?*

***21b****. Author comment :A: For what concerns run-off river power plants: there are a few of them, but over all their installed capacity is negligible with respect to reservoir system*

*R:Maybe it would be good to state that in the manuscript and not inly in the reviewer reply?*

21a. It seems from this comment that is not clear from the text what was present in Calliope_Base and what has been enhanced in Calliope_Hydro, and in which sections of the manuscript this has been explained.

We added the following lines to make this more clear:

Line 274-277: *“Section 2.1 and 2.2 describe the modelling methodology adopted for enhancing Calliope_Base into Calliope_Hydro, taking into consideration the four points highlighted at the beginning of this chapter. Section 3 presents the case study and how the new methodology is applied to it, while section 4 presents and comments the results.”*

Line 279-284: *“In the current version of Calliope, here referred to as Calliope_Base, the modeler can make use of a series of predefined supply and conversion technologies archetypes (i.e. Supply, Supply +, Conversion, Conversion +, Storage) and the standard way of modelling reservoir hydropower is by means of a Supply + technology, a technology that allows a flow of energy to enter the system based on a determined resource availability, and that can have a storage integrated, (like for example a PV power plant with a Battery Energy Storage System). Such existing technology archetypes are used here to better model reservoir hydropower.”*

Line 285-286: *“In this subsection we outline how we delt with points (I) time-dependent water inflow patterns in the basin due to hydrological processes and (II) water inflow supplied by upstream reservoirs.”*

Line 317-318: *“In this subsection we outline how we delt with points (III) dam maximum and minimum operational level and related water release constraints and (IV) evaporation losses dependent upon the reservoir surface.”*

21b. We agree, it has been inserted. Lines 406-408: *“Run-off river plants exist in the considered study area, but their installed capacity is negligible with respect to the considered reservoir system, and for this reason left out of the scope of the modelling effort carried out in this work.”*

***25.*** *Author comment : In my opinion not only the amount of electricity potential, but also location and sizes of reservoirs should be presented. I am still lacking an explanation why the SAPP region has been selected. The authors then give a lot of explanation about the power pools. But wouldn’t be the type of hydropower and typical hydropower operation schemes, turbine/dam types more relevant for the reader*

*R: Thanks for adding the table, that help a lot. But I am still missing an explanation why the SAPP region has been selected or why the other thing that it is a good area for a case study? Or was it simply chosen due to data availability? That’s also fine, but should be mentioned.*

Line 408-414: *“The ZRB and the SAPP were selected as case study because they represent an extremely suitable ground for testing our approach, different countries with different electricity generation mix, interconnected with each other and with a relevant river basin flowing through many of the involved countries, with dams distributed among them. It is an area widely studied by the scientific community in terms of Water-Energy Nexus [78] and in addition to that, the participation of the authors in the DAFNE project (https://dafne.ethz.ch/) made data easily accessible making in it the final decision for the case study.”*

***26.*** *Author comment: Line 307: So there are no environmental regulations / minimum flow regulations that the hydropower operators have to balance as well? There are minimum flow regulations only in ITT, but was not implemented as out of the scope of this work*

*R: Then I think it should be mentioned that they are out of scope.*

Correct, we inserted it. Line 486-487: *“For what concerns environmental and minimum flow regulations, only ITT has minimum flow regulations, but they were not implemented as out of the scope of this work.”*

***28.*** *Author comment: Line 309: What is a large reservoir? Until now no information about reservoir size was provided. Does the “the mean annual river flow” refer to the river section in which the reservoir is located? A large dam is a dam higher than 15 m as defined by the World Commission on Dam*

*R: How should the reader now that you refer to the definition by the “World Commission on Dam”*

Line 477: *“The four dams are considered “large dams” according to the World Commission of Dams [96], being higher than 15 m.”*

***32.*** *Author comment: Thanks for point out that you provided the hydrological data. But maybe then state it in the manuscript as you did in the reviewer reply. “ “Inflow data from 1986 to 2005 are extracted from the ADAPT project”[1] using the following gauging stations: Kafue Hook Bridge, Victoria Falls IN, Great East Road Bridge, and Mangochi. [1]» As for now in Line 425 it only says: Inflow data(the SW block in Figure 5) are extracted from the ADAPT project [87].*

Lines 477-481: *“Table 2 reports details on the four dams. Notably, the dams are located in different countries and may be linked with each other. Inflow data (the SW block in Figure 5) from 1986 to 2005 are extracted from the ADAPT project [97] using the following gauging stations: Kafue Hook Bridge, Victoria Falls IN, Great East Road Bridge, and Mangochi.”*
