# Supplementary material for: Advancing the representation of reservoir hydropower in energy systems modelling: The case of Zambesi River Basin
Source: PLoS One. 2021 Dec 2;16(12):e0259876. doi: 10.1371/journal.pone.0259876 (PMC8638992; doi:10.1371/journal.pone.0259876)
Supplement: Attachment — Submitted filename: Response to Reviewers_3rdRev.docx [file pone.0259876.s005.docx]

**Ref: PONE-D-20-40043**

**Title: Advancing the representation of reservoir hydropower in energy systems modelling: the case of Zambesi River Basin**

**Journal: PLoS One**

**Response to Reviewers:**

**EDITOR COMMENTS:**

***1.*** *Reviewer 2 suggests to shorten the introduction, as it is longer than the methods described in section 2. Although I agree that the introduction is quite long and could be shortened, I recognize that section 3 about the case study also describes methods and the methods description is, thus, longer than perceived by that reviewer. I suggest to only shorten the introduction to some extent if it doesn’t change the content anymore, as the introduction has already undergone a major revision in previous rounds.*

We agree that the introduction is quite long, we reduced the detail of the description of the literature reviewed in chapter 1.2, without hindering the sense or changing the content. Since, as agreed, the scope of the work is not a literature review, we believe this modification does not subtract to the overall quality and does not reduce the scope of the work. For what concerns chapter 1.3, we did not reduce the detail of description because we believe that it is instrumental to the introduction of the scope of the work to fully describe representation of hydropower in existing energy models.

***2****. In Table 1, please indicate the order of the latitude and longitude for the coordinates column.*

This has been fixed.

***3.*** In Figures 6 to 9, please remove the figure titles. They are redundant, given the figure captions.

This has been taken care of.

***4.*** *Please deposit your data and code in a static repository with a doi. GitHub is not a proper repository for such a purpose. You can, for example, use Zenodo to archive a GitHub repository, make it static, and assign a doi.*

Thanks for suggesting this. The github repository has been deposited in Zenodo and the relative doi is: https://doi.org/10.5281/zenodo.5242926

**REVIEWER #1 COMMENTS:**

***1.*** *As I stated in my previous reports, the article requires professional proofreading before it can be published*

We did our best to further improve the language.

***2.*** *Your response number 10: the response still does not makes sense to me. The authors state, “The nature of the power system, either single or open energy market is not relevant for the current application, nor hindering the validity of the obtained results.” Most African countries are not interconnected as a single market, and simulating them as a single market where electricity generation and use is driven by costs and prices is unrealistic and will undoubtedly have implications for the results. The authors mention in line 525 that the simulation error is because of this market assumption; so, this assumption has implications for the results. Models should represent reality to an acceptable level, and getting the type of market right is necessary for the present context—this major limitation of the case study application needs to be highlighted to the reader more clearly. You should add new text to discuss the implications of this limitation.*

This limitation has been highlighted and discussed. Lines 630-639.

*“Another limitation of the proposed case study, even though common in similar studies [94,95], is the assumption of a single market for the entire power pool. Each of the modelled countries, even though exchanging with the neighboring ones, is not yet part of a single SAPP energy market. This assumption partially influences the results in terms of exchanged power, and in turn, of produced power. The model optimizes on the minimization of the overall cost of operating the entire system, in this way the energy mix of a country is influenced by its possibility to fulfill the energy needs of another country. This results in a higher use of technologies with lower operation costs, that are employed to cover the demand of the countries that have more costly technologies in their production park, inside the limits of transmission capacity. Nonetheless, this does not hinder the value of the proposed methodology, that is still demonstrated to be valid by the case study.”*

**REVIEWER #2 COMMENTS:**

***1:*** *Thanks a lot for removing the Literature review section and adding the section “Representation of hydropower in energy modelling”. This section is much better. Thanks.*

*However, when it comes to the introduction, section “1.4. Aim of the work” still needs to be improved. Looking at the track changes I see only 2 small changes despite the comments for the editor: “2. Likewise, the contribution of your study remains unclear. It even seems more unclear than before, as the scope of the study was clarified to be much more limited than it seemed to be”, and reviewer 1: “7. Aim of the work: the scope of the work is to improve the representation of hydropower in an open-source energy modelling tool. That is fine, but how does this compare to integrated water-energy modeling approaches? Should the science and practice drop the idea of integrated water-energy modeling and try to improve hydropower representation in energy models? What I am missing here is a bridge between the literature review and the study contribution. It would be best to highlight how your work provides added value compared to previous approaches, including integrated water-energy modeling. This is a major issue that needs to be addressed. If the authors cannot find an added value of their proposed approach compared to comprehensive water-energy modeling, they should focus the paper around the case study rather than the modeling approach”*

*You mentioned that you changed something in lines 138-150. This is however not in the “Aim of the work chapter”. In other words, the link between 1.3 and 1.4 should still be improved.*

We agree that was missing a connection between chapter 1.3 and 1.4, and chapter 1.4 was starting abrupted. We have inserted now lines 191-198:

*“As observed in chapter 1.2 and 1.3, model integration between energy and hydro models results in drawbacks in terms of computational time and non-replicability, among others. On the other hand, while only-energy models, and in particular open-source ones, are meant to build in the direction of solving such issues, they lack in proper characterization of hydrological phenomena and hydropower production. For these reasons, the aim of this work is to provide a methodology for improving the characterization of reservoir hydropower in open-energy-modelling. Through this methodology we aim at reaching an advance in the energy modelling science, by learning from the integrated modelling experience analyzed in chapter 1.2, and overcoming the highlighted limits thanks to the advantages brough about by the open-modelling framework.”*

And we believe this lines give now the proper structure to the chapter.

***2.*** *On another note, the authors sate: “Please consider that a literature review is not the goal of the paper, and the attention of the reader should be focused on the methodology.”*

*However, Lines 39-244 are dedicated for the introduction, while lines 246 – 376 are for the methods.*

Thanks for highlighting this, measures for shortening the introduction have been taken. Please see answer to Editor’s comment #1 for a more detailed response.
